# Strengthening HIV surveillance in the antiretroviral therapy era: rationale and design of a longitudinal study to monitor HIV prevalence and incidence in the uMgungundlovu District, KwaZulu-Natal, South Africa

**DOI:** 10.1186/s12889-015-2179-2

**Published:** 2015-11-20

**Authors:** Ayesha BM Kharsany, Cherie Cawood, David Khanyile, Anneke Grobler, Lyle R. Mckinnon, Natasha Samsunder, Janet A Frohlich, Quarraisha Abdool Karim, Adrian Puren, Alex Welte, Gavin George, Kaymarlin Govender, Carlos Toledo, Zawadi Chipeta, Lycias Zembe, Mary T Glenshaw, Lorna Madurai, Varough M Deyde, Alfred Bere

**Affiliations:** Centre for the AIDS Programme of Research in South Africa (CAPRISA), Doris Duke Medical Research Institute, Nelson R Mandela School of Medicine, University of KwaZulu-Natal, 2nd Floor, Private Bag 7, Congella, 4013 Durban South Africa; Epicentre AIDs Risk Management (Pty) Limited, P O Box 3484, Paarl, 7620 Cape Town South Africa; Centre for HIV and STIs, National Institute for Communicable Diseases, National Health Laboratory Service (NICD/NHLS), Johannesburg, South Africa; South African Centre for Epidemiological Modelling and Analysis (SACEMA), Stellenbosch, South Africa; Health Economics and HIV and AIDS Research Division (HEARD), University of KwaZulu-Natal, KwaZulu-Natal, South Africa; Centres for Disease Control and Prevention (CDC) Atlanta, Atlanta, USA; Centres for Disease Control and Prevention (CDC), Pretoria, South Africa; Global Clinical and Virology Laboratory, 11 Dan Pienaar Place, Amanzimtoti, South Africa

## Abstract

**Background:**

South Africa has over 6,000,000 HIV infected individuals and the province of KwaZulu-Natal (KZN) is the most severely affected. As public health initiatives to better control the HIV epidemic are implemented, timely, detailed and robust surveillance data are needed to monitor, evaluate and inform the programmatic interventions and policies over time. We describe the rationale and design of the HIV Incidence Provincial Surveillance System (HIPSS) to monitor HIV prevalence and incidence.

**Methods/Design:**

The household-based survey will include a sample of men and women from two sub-districts of the uMgungundlovu municipality (Vulindlela and the Greater Edendale) of KZN, South Africa. The study is designed as two sequential cross-sectional surveys of 10,000 randomly selected individuals aged 15–49 years to be conducted one year apart. From the cross sectional surveys, two sequential cohorts of HIV negative individuals aged 15–35 years will be followed-up one year later to measure the primary outcome of HIV incidence. Secondary outcomes include the laboratory measurements for pulmonary tuberculosis, sexually transmitted infections and evaluating tests for estimating population-level HIV incidence.

Antiretroviral therapy (ART) access, HIV-1 RNA viral load, and CD4 cell counts in HIV positive individuals will assess the effectiveness of the HIV treatment cascade. Household and individual-level socio-demographic characteristics, exposure to HIV programmatic interventions and risk behaviours will be assessed as predictors of HIV incidence. The incidence rate ratio of the two cohorts will be calculated to quantify the change in HIV incidence between consecutive samples. In anticipation of better availability of population-level HIV prevention and treatment programmes leading to decreases in HIV incidence, the sample size provides 84 % power to detect a reduction of 30 % in the HIV incidence rate between surveys.

**Discussion:**

The results from HIPSS will provide critical data regarding HIV prevalence and incidence in this community and will establish whether HIV prevention and treatment efforts in a “real world”, non-trial setting have an impact on HIV incidence at a population level. Importantly, the study design and methods will inform future methods for HIV surveillance.

## Background

The HIV epidemic has reached an unprecedented scale in southern Africa, with South Africa having the highest number of infected individuals worldwide. The South African National HIV Prevalence, Incidence, Behaviour and Communication Survey of 2012 estimated that amongst individuals in the 15–49 year age group, the overall HIV prevalence was 18.8 % [95 % Confidence interval (CI) 17.5–20.3]. However, HIV is unevenly distributed across all provinces and the worst affected province is KwaZulu-Natal (KZN) with a prevalence of 27.9 % (95 % CI 25.2–30.8) compared to the Western Cape which has a prevalence of 7.8 % (95 % CI 5.5–10.9) [1]. In South Africa, young women acquire HIV at least 5 to 7 years earlier than their male peers and bear a disproportionate burden of HIV [2, 3]. In 2012, the HIV prevalence in young women in the 15–24 year age group was 11.4 % (95 % CI 9.8–13.2) compared to 2.9 % (95 % CI 2.1–3.9) in young men in the same age group [1].

In addition to household surveillance, the South African National Department of Health (SA DOH) conducts annual anonymous seroprevalence surveys among pregnant women utilizing public sector health care facilities across South Africa. These surveys have captured increases in HIV prevalence over time from 0.8 % in 1990 reaching 30.2 % in 2010 and stabilizing to 29.5 % in 2011 and 2012 [3, 4]. Similar to the household data, the data from pregnant women mask geographical variations and in 2012 the HIV prevalence in KZN was 37.4 % (95 % CI 36.0 – 38.7) in contrast to 16.9 % (95 % CI 13.8 – 20.5) in the Western Cape. Additionally, seven of the nine districts within KZN have recorded antenatal HIV prevalence above 38.0 % with the uMgungundlovu district having a prevalence of 40.7 % [5]. These trends in HIV prevalence continue both provincially and nationally [5].

Prospective cohort studies in KZN have demonstrated that young women are particularly vulnerable to HIV [1, 3]. Across urban and rural areas, high HIV incidence rates have been observed: 6.5/100 women years of observation (wy) (95 % CI 4.4–9.2) in Vulindlela, 6.4/100 wy (95 % CI 2.6–13.2) in Durban [6], 14.8/100 wy (95 % CI 9.7–19.8) in Ladysmith, 6.3/100 wy (95 % CI 3.2–9.4) in Edendale, and 7.2/100 wy (95 % CI 3.7–10.7) in Pinetown [7]. Recent data from the CAPRISA 004 tenofovir gel trial has shown that HIV incidence rates in 18–40 year old urban (Durban) and rural (Vulindlela) women in the placebo gel arm was 9.0/100wy (95 % CI 5.3–14.3) and 9.1/100wy (95 % CI 6.6–12.3) respectively [8].

Using the Limiting-Antigen Avidity Assay (LAg-Avidity EIA) of measuring HIV incidence from cross sectional sampling, at the national level, HIV incidence measures show differential HIV transmission dynamics. In 2012, HIV incidence was 2.3 % (95 % CI 1.8–2.7) in women aged 15–49 years, which was 1.7 times higher than the 1.21 % (95 % CI 0.9–1.5) in men. In 15–24 year old women, HIV incidence was 2.5 % (95 % CI 2.0–3.0), which was more than four times higher than the 0.6 % (95 % CI 0.5–0.7) in men of the same age [1]. These rates suggest that HIV remains exceptionally high and the underlying dynamics of transmission within communities is not well understood.

To counter these high rates, the South African government launched the national HIV prevention and treatment campaign in 2010 [9], which aimed to increase HIV counselling and testing (HCT) and antiretroviral therapy (ART) uptake, rigorous implementation of prevention of mother-to-child transmission (PMTCT) of HIV, roll out of voluntary medical male circumcision (VMMC) and the provision of post exposure prophylaxis (PEP) [10]. These programmes have resulted in some degree of success [11], however, providing optimal coverage to prevent further spread of HIV requires a nuanced understanding of the underlying drivers of HIV transmission in conjunction with programmes that are in place across the region. Strengthening the effectiveness of ART roll out requires advanced monitoring of heterogeneous outcomes that are influenced by variable rates of uptake, adherence and retention in follow-up. In addition to ART [11] and early ART initiation [12], known as ‘treatment-as-prevention’ (TasP) [13–16], many biomedical interventions are undergoing evaluations and could be implemented in the near future. These include vaginal microbicides [8]; oral [17–19] and long-acting injectable [20] pre-exposure prophylaxis (PrEP); and structural measures such as conditional cash transfers (CCT) [21, 22], all of which are crucial components of a comprehensive HIV prevention strategy that could potentially have an impact on HIV incidence rates over time [23].

Given the imperative to scale-up HIV treatment, biomedical and structural interventions, there is a tangible need for independent mechanisms to monitor and determine which programmes have the capacity to alter epidemic trajectories in different geographical areas. In addition, as HIV prevention interventions become increasingly available, demand for access and distribution to those in highest need is a challenge for HIV programmers. Thus, better monitoring of HCT programmes on service uptake, linkage to HIV care and treatment, transmitted and acquired ART drug resistance and co-morbidities are crucial for the associated benefits on individuals and population levels [24, 25]. Measuring HIV prevalence trends over time is a major part of national and provincial strategic AIDS plans, however, these become difficult to interpret because of increasing survival over time amongst individuals on ART [26, 27]. This survival effect could limit the usefulness of using only HIV prevalence data for surveillance of new infections. Therefore, there is a need to measure HIV incidence to improve surveillance systems as it provides a more sensitive method to monitor trends in heterogenous epidemics [28].

Measuring HIV incidence is, however, challenging. HIV incidence measured by the traditional follow-up of cohorts of HIV-uninfected individuals or estimated using modeling methods have their limitations [28]. Laboratory-based assays such as the LAg-Avidity EIA can be used to generate cross-sectional incidence data and advances in the use of such assays has been recognized [1]. However, these assays require validation in different populations including geographical regions and the extent of ART use [29].

In order to sharpen and focus on delivering high quality HIV services, there is a greater need to better understand HIV transmission sexual networks that contribute to local HIV diversity. Phylogenetic analysis [30–33] and geospatial mapping [34, 35] of HIV-1 sequences are newer tools which have substantially advanced our ability to track the epidemic and intervene to prevent new HIV infections [36]. These tools provide an opportunity to identify spatial variation at regional, urban/rural, district and sub-district levels and whether HIV “hotspots” are clustered in relation to structural, sociodemographic and behavioural factors. Furthermore identifying HIV transmission chains within social sexual networks would provide empirical evidence for understanding the dynamic heterogeneity of HIV infection which to a significant degree is often masked at a country level [37]. It is also important to focus on “locations” [38] to get a better understanding of what’s driving this heterogeneity so that prevention and treatment efforts could be tailored and implemented within discreet and broader communities; and target key populations and high transmission areas (HTA).

We hypothesized that the intensified HIV programmatic interventions, prevention and treatment efforts of the KZN Provincial Department of Health (DoH), together with its implementing partners, would lead to reductions in HIV incidence among men and women in this region.

## Aim

The aim of this study is to establish a population-level HIV incidence Provincial Surveillance System (HIPSS) in a household-based representative sample of men and women in rural and peri-urban KZN and to use the data to inform the local and national DoH on the design and methodology of future HIV surveillance programs.

## Objectives

The primary objective is to:

- assess the impact of programmatic intervention efforts in a “real world”, non-trial setting on HIV incidence.

The secondary objectives are to:

- estimate the prevalence of HIV, CD4 cell counts in HIV infected individuals and proportion on ART and ART naïve with detectable and undetectable HIV-1 RNA viral load;
- measure prevalence of pulmonary tuberculosis (TB), sexually transmitted infections (STI) and hepatitis B and C infections;
- measure HIV incidence trends over time;
- compare cohort measurement of HIV incidence with those obtained from cross-sectional sampling and LAg-Avidity EIA;
- estimate community HIV-1 RNA viral load;
- estimate the spectrum of ART resistance in prevalent and incident HIV infections;
- geospatially map HIV infections in relation HIV-1 RNA viral load to identify specific clusters of infections and whether these contribute to enhanced HIV transmission;
- characterize HIV-1 genetic diversity in this populations and identify HIV transmission networks as sources of infection;
- identify risk factors for HIV incidence at the individual, household, and community levels.

## Methods

### Study setting

**Fig. 1:**
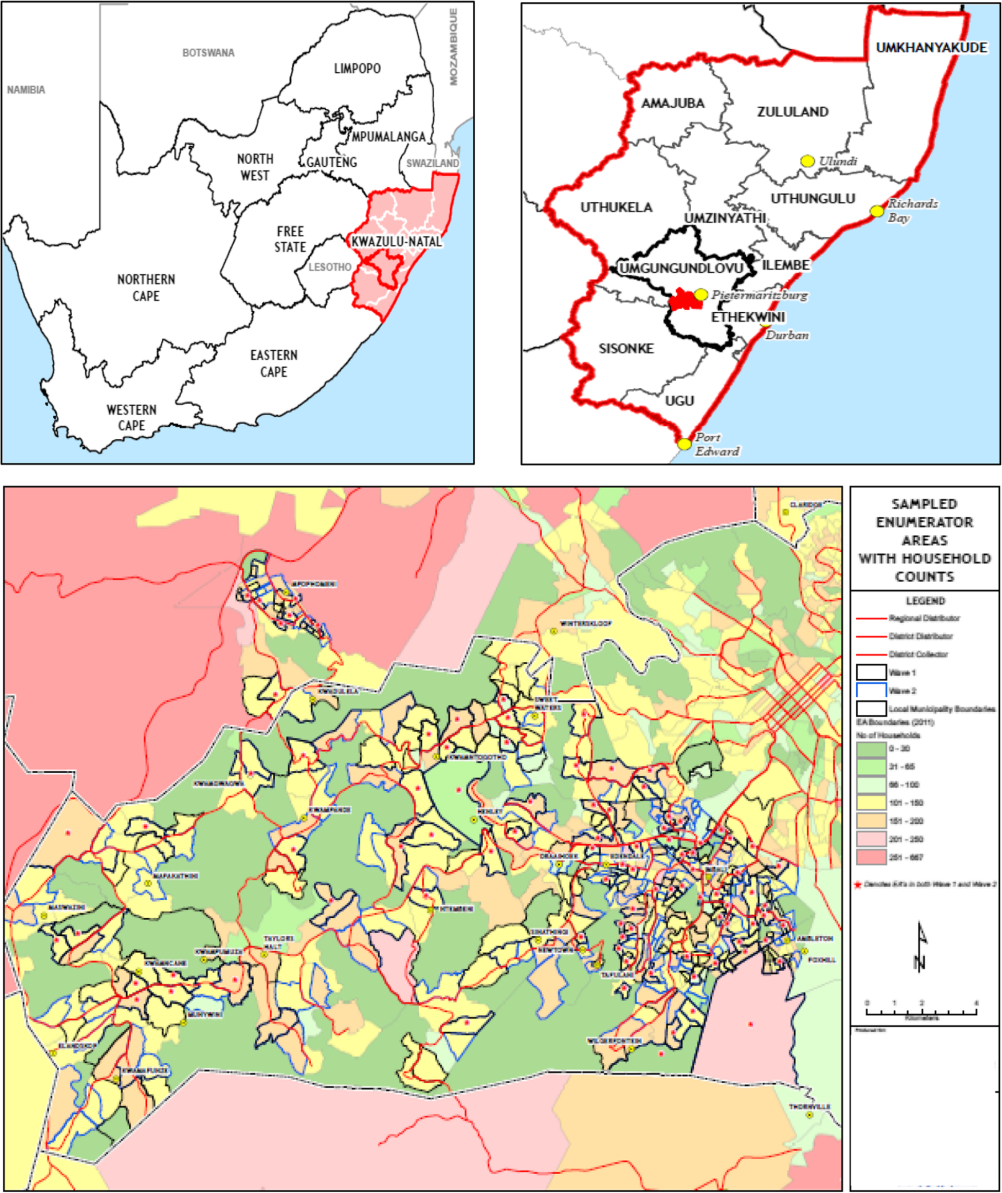
Location of Greater Edendale and Vulindlela study area in KwaZulu-Natal, South Africa

This study will be carried out in central KZN in Vulindlela and Greater Edendale, two of the seven sub-districts in the uMgungundlovu (Fig. 1 – Map of the study area) Municipality. The region incorporates rural traditional settlements or farmlands through to informal and peri-urban living.

Vulindlela is a rural community with a population of just over 150,000 predominantly Zulu speaking people. The majority of the land belongs to the traditional authority through the Ingonyama Trust and is made up of nine wards, of which five are under the traditional leadership and four are under the ward counsellors of the local government municipal system. The Greater Edendale area with a population of about 210,000 people is the second largest peri-urban area within KZN and is the main economic hub within the uMgungundlovu district. It is linked to Vulindlela by a dual carriage way known as the Edendale Corridor. This route serves as the connection between various outlying rural areas to the north of Pietermaritzburg. Much of the Greater Edendale Area is developed with both formal and informal housing, supported in some areas by ancillary land uses and facilities.

There are 16 primary health care clinics (PHC) in the area, within which trained nurses provide much of the health care. The PHCs are supported by three district hospitals that cater to people in the western area of KZN, including the study area. In addition, there are about 60 community-based organizations in the district representing a variety of civic interests such as youth, women, religions and political parties. Several of these organizations are currently providing HIV prevention and home-based care services.

### Study design

**Fig. 2:**
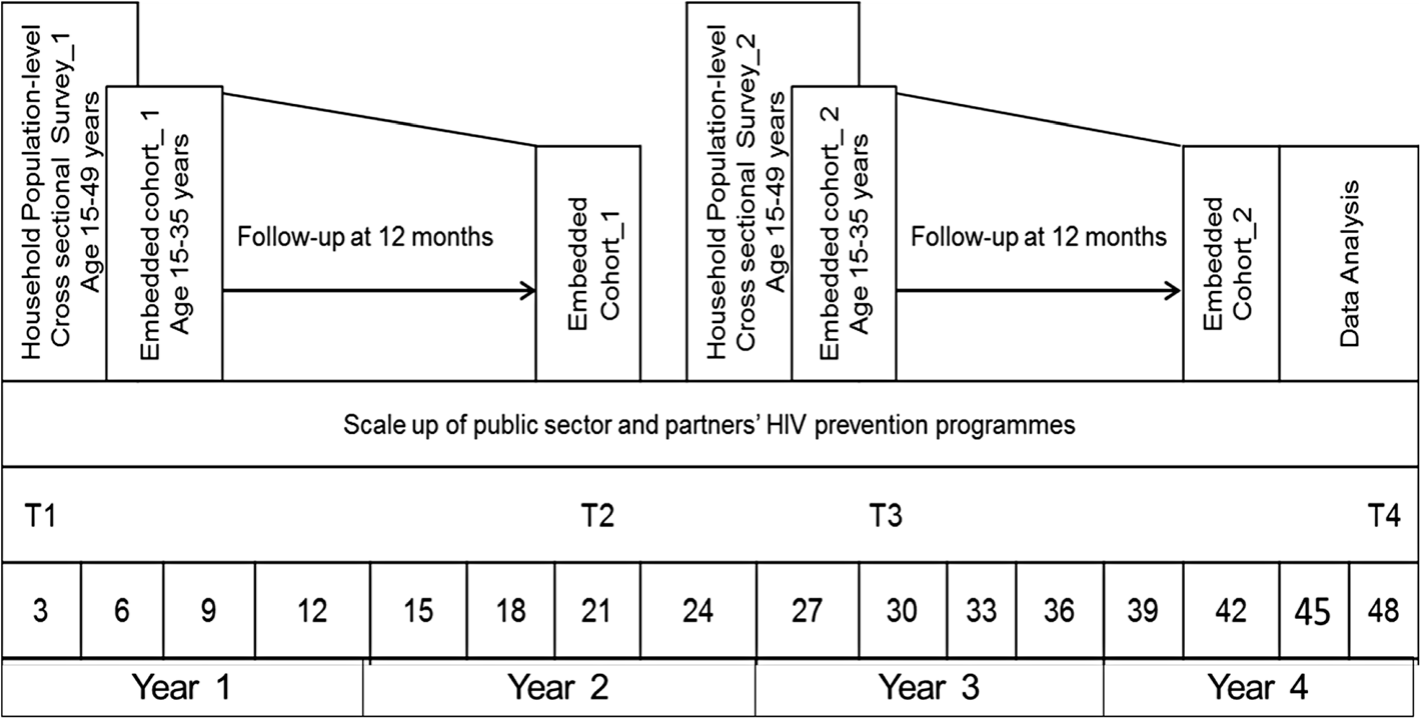
Study design and timelines

This study will establish two sequential, representative, cross-sectional household surveys of 10,000 individuals per round. The cross-sectional surveys will enroll individuals 15 to 49 years of age. Sequential embedded cohorts of HIV negative individuals between the ages of 15 and 35 years will be followed up prospectively and assessed for HIV infection after 12 months. A schematic diagram representing the overall study design is shown in Fig. 2.

### Study population

According to national census data [39, 40], the study area has an estimated 95,641 households with an estimated total of 367,906 individuals. Of these, 176,418 (48.0 %) are males and 191,515 (52.0 %) females. A total of 217,278 (59.1 %) are in the age range of 15–49 years and 164,302 (44.7 %) are in age range of 15–35 years.

### Sampling strategy

A two-stage cluster-based sampling of enumeration areas (EA) will be used to randomly select households. The sampling frame has been triangulated from the 2011 Census [40], the 2007 Community survey data (StatSA Community Survey) [41], and aerial imaging of dwellings supplied by the 2011 midyear estimates of Geo Terra Image (GTI). These will determine the number of households at an EA level.

EAs will be drawn randomly and within each EA, households will be drawn systematically with a random start in a serpentine pattern. Study staff will identify households and use a Global Positioning Systems (GPS) receiver to record the geographic coordinates of each randomly selected household. If households are not sampled for reasons of abandonment, refusal to participate, or if the members are away for an extended period of time, the household on the right side of the selected house, when facing its entrance, will be used as a replacement. All replacement households will be authorized by a supervisor. Sampling will continue until 10,000 households have been enrolled.

Once a household is selected, a handheld personal digital assistant (PDA) will be used to compile a list of all the individuals residing in the household. All individuals who meet the eligibility criteria will be numbered and the PDA will select one of these individuals at random to be included in the study. Only one individual per household will be enrolled. Should the selected individual refuse to participate the next randomly selected individual may be enrolled in the study. Should this second individual also refuse the household will be replaced as described earlier.

### Eligibility criteria

Men and women aged 15 to 49 years who reside in the selected household, who are willing to participate and provide written informed consent (or parental consent/child participant assent if < 18 years of age) in either English or Zulu, and are willing to undergo all study procedures including provision of clinical specimens (peripheral blood, urine, sputum-if indicated and for females, self-collected vulvo-vaginal swabs) will be eligible. Those who are non-residents of the household, who refuse to participate and/or provide samples, who cannot consent, are mentally challenged, or who plan to move within 12 months will be excluded. The longitudinal follow-up will include all HIV negative enrolled individuals 15 to 35 years of age from the cross-sectional survey.

### Statistical considerations and sample size calculation

The sample size of this study provides 84 % power to detect a 30 % reduction in HIV incidence rate at a 5 % significance level, given an HIV prevalence of 20 %, loss-to-follow-up of 15 % per annum and an initial HIV incidence rate of 3 per 100 person years.

To compare the changes in HIV incidence over time, the HIV incidence rate will be calculated for each cohort. The incidence rate ratio of the two cohorts will be calculated to quantify the change in HIV incidence between the two time periods. The assumptions for various study outcomes have been summarized in Table 1.

**Table 1:** Number of enrolled participants required to achieve the targeted effective number of person years of follow-up

	Time point	
	T1 and T2	T3 and T4
Cross sectional survey (Baseline) (age range 15–49 years)		
Number at baseline	10,000	10,000
HIV Prevalence	20 %	20 %
Number expected to be HIV negative	8000	8000
Longitudinal follow-up cohort (age range 15–35 years)		
Design effect	1.1	1.1
Number of individuals in the HIV negative cohort	6400	6400
Assumed HIV Incidence rate	3 %	3 % reduced by 30 %
Loss to Follow-up adjustment	15 %	15 %
Number expected to be retained in cohort	5440	5440
Number of HIV endpoints assumed	163	114
Total number of endpoints assumed across both cohorts	277	
Probability of Correct Inference	84 %	84 %

T1 = Cross sectional Survey 1

T2 = Embedded cohort 1

T3 = Cross sectional Survey 2

T4 = Embedded cohort 2

## Study procedures

### Recruitment

Study staff will approach the randomly selected households, make appropriate introductions, identify the head of household or designee and provide study information. A household composition form will be completed to capture age, gender, and basic socio-demographic profile of all usual household members, including questions on household economic status, food security, access to health services, health status and changes in composition and deaths in the last 12 months. The PDA has been programmed to randomly select one individual who meets the eligibility criteria and that person will be asked to participate in the study. Those who agree to participate will be prepared for baseline assessments and enrolment.

### Baseline and follow up assessments

Eligible participants will receive study information; provide consent (or assent), biometric fingerprints, and locating information before enrolment into the study. Each participant will be assigned a unique study number that will be linked to the structured questionnaire to be administered by study staff using the PDA. Study staff with training in phlebotomy will collect peripheral blood sample (25 ml). Participants will be guided to collect sputum (if indicated based on the presence of any signs and symptoms of TB), 10–20 ml of first-pass urine sample (males), and self-collected vulvo-vaginal swab samples (females).

#### Study staff will administer questionnaires to obtain

- demographic variables including age, gender, marital status, socioeconomic status, occupation, employment and educational status,
- psycho-social variables including knowledge/motivational issues, capabilities, social norms and affective states/situational contexts related to sexual risk behavior and indicators of depression;
- behavioural variables including partner characteristics, number, type (regular/casual), and concurrency of sex partners, condom use, alcohol use, knowledge of own and sex partner(s) HIV status, engagement in transactional sex and exposure to intimate partner violence;
- HIV status variables, including HIV testing history and date of last HIV test, HIV test results and linkages to HIV medical and psychosocial care, ART use, exposure to and treatment for TB and STI;
- contraceptive use (females only); and
- male circumcision status (males only), including whether circumcised, and acceptability and access to VMMC. Exposure to any informational, educational, behavioural and/or biomedical prevention, treatment and psychosocial support programmes for HIV through the DoH, community organisations and/or PEPFAR partners in the district will be captured.

Participants completing the baseline survey will be informed that they might be contacted for the follow-up survey in approximately 12 months. Participants, eligible for the cohort will be contacted. Study staff will verify the participant’s identity and administer a follow-up questionnaire using the PDA and collect samples for follow-up assessments. Any refusals and lack of retention for any reason will also be captured.

### Access to health care

HCT services will be made available to participants and family members, with referral systems to access care, support and treatment. Participants with signs and symptoms of STIs and/or TB will receive a symptom specific referral note to district PHC services for care and services. A key component of this survey is related to the provision of HIV test results to participants. All participants will be provided with a barcoded card and informed that they have an opportunity to access their HIV test results from the samples that have been collected from the survey. The laboratory results for HIV serology, CD4 cell count, HIV-1 RNA viral load, STIs and TB will be sent to all PHC clinics in the study area for participants to access using their barcoded card. In collaboration with the DoH, all PHC clinic staff have been trained to receive study participants, provide results with appropriate counselling and follow-up care. The number of individuals referred will be recorded to determine the number of people accessing these services.

## Outcomes and analysis

The primary outcome will be HIV incidence measured through prospective follow-up of the cohorts at 12 months following enrolment.

### Analysis of the primary objective

HIV incidence will be estimated at two time points in this household-based representative sample of men and women. Only participants who test HIV negative at enrolment will be included in this analysis. The person-years in follow-up will be calculated as the difference between the date of the enrolment HIV test and the 12-month HIV test. The date of HIV infection will be assumed to be the midpoint between the last HIV negative test and the first HIV positive test. To achieve a population HIV incidence rate, the total number of new HIV infections will be divided by the total number of person-years. Incidence rates will be presented in conjunction with 95 % CI, and stratified by age group and gender. The incidence rate ratio (IRR) of the two cohorts will be calculated to quantify the change in HIV incidence between the two time periods, including 95 % CI calculated using Poisson approximations (for incidence) or the F-distribution for IRR. Comparisons of HIV incidence from the two cohorts over two time points will assume a Poisson distribution of new HIV infections in the follow-up time in each cohort.

### Analysis of secondary objectives

- **HIV linkage to care and treatment cascade:** The proportion of HIV-positive men and women in HIV care, on ART by self-report and laboratory measurement of ARV, and with viral suppression or detectable virus will be determined. Logistic regression will be used to assess any differences in proportions of men and women accessing ART. These analyses will be stratified by CD4 cell counts and adjusted for potential confounders.
- **Associations of new HIV infections:** The association between new HIV infections and several baseline predictive variables will be assessed. Survival analysis methods will be used to calculate hazard ratios in follow-up time varies within the sample, or multivariate logistic regression if follow-up durations are similar. Variables in the baseline survey to be included in the analysis will be demographic information (age, gender and area of residence), psycho-social variables, and partner characteristics, situational factors associated with sexual practices, sexual behavior, HCT, VMMC, condom use, and exposure to behaviour change information, training and communication. Household-level variables will also be used as predictors of HIV acquisition.
- **Prevalence and incidence of STIs, Hepatitis B and C:** The prevalence of STIs including gonorrhoea, chlamydia, trichomoniasis, herpes simplex virus type 2 (HSV-2) antibodies, syphilis, and human papillomavirus (HPV) infection will be measured. Hepatitis B and C prevalence will be measured. All estimates will include 95 % CI and stratification by age and sex. The incidence of STIs and Hepatitis B and C will be measured in participants who test negative for these infections at enrolment. Person-years of follow-up, date of infection and incidence rates will be calculated as above for HIV.
- **Prevalence and incidence of pulmonary TB:** The case definition of TB will be a positive result on the automated diagnostic test, the GeneXpert MTB/RIF for the identification of *M. tuberculosis* DNA and resistance to rifampicin by nucleic acid amplification technique. Prevalence by age and sex will be calculated for both HIV positive and negative participants. Only participants who tested negative for TB at enrolment will be included in TB incidence measures. Incidence rates, 95 % CIs, date of infection, and stratifications will be done as for other infections.
- **Performance of laboratory assays for HIV incidence measurement:** The LAg-Avidity EIA assay derived incidence will be compared to the incidence rate derived from the prospective cohort using parameters recommended for the assay. We will use the latest available data on false recent rates (FRR) of the chosen algorithm (using data from CDC and the Consortium for the Evaluation and Performance of HIV Incidence assays) to estimate the locally applicable FRR [42]. Assay-based HIV incidence will be calculated as the number of recent infections divided by the population at risk (those testing HIV-negative plus those recently seroconverting), annualized by multiplying by 365 divided by the estimated length of mean seroconversion duration for the assay (130 days for the LAg-Avidity EIA assay; 95 % CI 118–142 days).
- **Measurement of community HIV viral load:** The mean viral load of a community will be used as an indicator of ART uptake, coverage and continued HIV transmission. The community viral load will be calculated as the arithmetic mean of viral load measures in HIV seropositive participants and will provide a baseline measurement for determining impact of programmes on increasing ART use and viral suppression. Undetectable viral load will be counted as 50 copies when calculating the mean.
- **Prevalence and incidence of transmitted HIV drug resistance:** The 1.3 kb fragment of the HIV-1 pol gene will be amplified for the detection of genotype resistance mutations. The prevalence of drug resistance will be determined by calculating point prevalence with 95 % CIs according to threshold levels of low (< 5 %), moderate (5–15 %) and high (> 15 %) in individuals with prevalent HIV infection at baseline. Results will be stratified by self-reported ART status and history. In individuals with incident infection during follow-up, the incidence of transmitted resistance will be measured.
- **Geospatial mapping of HIV infections:** Using the household GPS co-ordinates, prevalent and incident HIV infections will be mapped to identify location of infections. Using a two-dimensional Gaussian kernel, prevalent and incident infections will be mapped across continuous geographical space. The Kulldorff spatial scan statistic (Bernoulli model) will be used to identify clusters of infections (*P* < 0.05) to determine any geographical heterogeneity.
- **Identifying transmission linkages constituting the sexual networks driving high HIV incidence:** The genetic diversity of the viruses in these populations will be used for the identification of transmission clusters. Phylogenetic trees will be generated using maximum likelihood methods and network analysis to characterize transmission clusters and their members, describe the network characteristics of these transmission chains and analyze the contribution of HIV clusters to new infections.
- **Identifying socio-demographic, biological and sexual risk behaviours:** Multivariable analyses of self-reported data will assess the extent of these factors and their contribution to HIV prevalence and incidence.

#### Ethical considerations

Extensive community engagement has been established with local stakeholders including traditional leaders from the community, service providers for health, social and economic development in this catchment area, including government and public and private organisations who provide services related to education, psycho-social support, socio-economic support as well as treatment. In addition, health-service providers from each of the PHCs in the district have been trained to provide appropriate care and management of participants referred to clinics. All stakeholders are provided with information on the South African HIV epidemic, availability and access to the governments HIV prevention and treatment programmes and the rationale for undertaking this study.

All study participants will provide written informed consent (or parental consent/child participant assent if < 18 years of age) in either English or Zulu prior to any study related procedures. Inclusion of individuals under the age of 18 years has been driven by the consistent finding that HIV prevalence and incidence are considerably high in this age group, particularly in females [6, 43]. Data on 15–24 year old prenatal women and those from the general population show that HIV prevalence and incidence remain higher in this age group compared to males in the same age group [1, 4]. These data underscore the need to continually monitor this sub-group. In addition, there is a precedent and a framework in South Africa for individuals’ ≥ 15 years of age to give informed consent for participation in sexual reproductive health research. Whilst the age of consent in South Africa for having an HIV test is 12 years, it is theoretically possible to include those < 15 years in this study; however, the ethical considerations related to confidentiality, anonymity and protection of children preclude their participation despite them being sexually active and at risk for HIV infection. Separate studies may be required for this sub-population.

The embedded cohort study sample is biased towards the younger population, where most HIV incident infections are observed; this is intended to enhance the efficiency of the study, rather than the inclusion of an older population where fewer incident infections are expected to occur. Persons with a stated intention to leave the study area indefinitely may be more easily lost-to-follow-up. Substantial attrition from these persons could reduce the power of the study, and ultimately jeopardize the ability of the study to detect differences in the key study outcomes.

## Discussion

Recent reports suggest that South Africa has made remarkable gains in the scale up of ART, leading to substantial reductions in mortality, increasing survival and HIV prevalence [11]. Parallel to these gains there have been a considerable decline in incident HIV infections. However, despite these gains, HIV incidence remains unacceptably high in this region [38]. The key question is what intensity and coverage of HIV prevention and treatment initiatives will be needed to significantly reduce HIV incidence and maintain effective control over the longer term to eliminate HIV infection as an important public health measure. As HIV transmission varies over time, place, and population group, understanding where prevalent and incident HIV infections are located geographically, and how they are being acquired (modes of transmission), has been important for monitoring temporal trends of the evolving pandemic, guiding the targeting of prevention efforts, assessing the impact of interventions, and planning for health care delivery needs [38, 44, 45]. This requires increased efforts for regular, systematic and cost-effective monitoring strategies.

In many countries, the extent and magnitude of HIV infection remains uncertain as a result of insufficient disease surveillance systems and diagnostic capabilities. This has improved dramatically through the development of HIV antibody testing, which has helped countries to identify emerging HIV epidemics, set up surveillance systems to better understand the modes of transmission, track and understand the natural history of infection and provide a reasonable picture of the evolving epidemic. South Africa has robust country level HIV surveillance systems to monitor HIV prevalence among pregnant women attending public sector primary health care clinics [4] and in the general population through the household surveys [1]. These have over time improved the reliability of national HIV prevalence estimates and provide robust information on the evolving epidemic at a country level. However, a nuanced understanding of the heterogeneity of the epidemic at the local level including in specific locations is limited, and would be highly beneficial to maximize on limited resources.

The establishment of this study will contribute to monitoring the uptake and impact of HIV prevention efforts and extend into overall health impact. As opposed to “efficacy”, which measures a program’s effect under highly controlled conditions, “impact” (or community-level effectiveness) is the effect of a program on a population level as measured by changes in incidence, prevalence, mortality, and/or other ultimate outcomes of interest such prevalence of TB, STIs and teenage pregnancy rates. As the KZN Provincial Government, PEPFAR partners and other local organizations are scaling-up intensive, multi-pronged prevention interventions including HCT, VMMC, and early treatment for HIV, it is important to collect localized and detailed information about the HIV response in a geographic area that has the ability to look more closely at associations between the scale-up of prevention efforts and changes in HIV incidence in a “real world”, non-trial setting at a population-level.

Many methods, other than prospective cohorts, to estimate HIV incidence have been developed in an attempt to provide a more sensitive and reliable indicator of the impact HIV prevention efforts. HIV incidence estimation however poses methodological challenges and on-going efforts are needed to strengthen our ability to accurately measure incidence. Therefore, this study has the potential to evaluate different laboratory assays including those that are newly developed.

The activities, aims, and outcomes of the HIPSS study would provide valuable information to the province to enable the implementation of the surveillance strategy across other districts and are aligned to the National and Provincial AIDS Strategic plan [10], UNAIDS 90–90–90 targets [45] and PEPFAR’s Determined, Resilient, AIDS-free, Mentored, and Safe (DREAMS) project [46] which collectively aim to address risk behaviours, gender based violence, enhance knowledge of HIV status, uptake of ART and reduce HIV transmission. The HIPSS study will be implemented in close consultation with the District Health Management team and so approaches and results identified as a priority will be aligned to the needs of the District, Provincial and National health systems. This is key to sustainability of HIV surveillance to monitor the epidemic over time.

Our study has some limitations. As with all survey data, there is the potential of underreporting of sensitive behavioural data. This is often due to a combination of poor recall, participants being untruthful, inadequacies in measurement instruments, social desirability bias, and sampling bias. The extent of underreporting is somewhat mitigated by the use of PDA-based interviews since the presence of the PDA enhances participants’ perceptions of privacy and thus increase responses to sensitive questions. Recall bias is also limited as some response categories act as triggers for follow-up questions.

However, setting up district level surveillance systems will provide detailed information about the HIV response in the specific geographic areas and the ability to look more closely at associations in scale-up of prevention efforts on changes in HIV incidence. While the South African National HIV Prevalence, Incidence, Behaviour and Communication Survey is extremely valuable to monitor the HIV epidemic in South Africa its large geographic coverage and cross sectional methodology makes it difficult to assess more localized incidence dynamics with any statistical power.

In summary, South Africa has one of the highest HIV prevalence and incidence rates in the world. The province of KZN with the highest infection rate in the country is taking a leading role in implementing a combination of programmes for HIV prevention and treatment at an unprecedented pace. It is vital to describe changes in the rate of new HIV infections resulting from implementation of these interventions. The HIPSS study described in this protocol seeks to document changes in HIV incidence and examine factors that may contribute to these changes so that other districts, provinces, countries and programs can develop and implement HIV surveillance strategies to rapidly monitor anticipated changes in HIV incidence.

## Regulatory approvals

The study protocol, informed consent and data collection forms have been reviewed with ethics oversight and approval from the University of KwaZulu-Natal Biomedical Research Ethics Committee (BF269/13) and the Centers for Disease Control and Prevention from the United States of America and in collaboration with the Department of Health, Province of KwaZulu-Natal (HRKM 08/14).

## Study status

Enrolling

## Abbreviations

ART: Antiretroviral therapy
BREC: Biomedical Research Ethics Committee
CAB: Community Advisory Board
CCT: Conditional cash transfers
CI: Confidence interval
DOH: Department of Health
DREAMS: Determined, Resilient, AIDS-free, Mentored, and Safe project
EA: Enumeration areas
GTI: Geo Terra Image
HCT: HIV counselling and testing
HTA: High transmission areas
KZN: KwaZulu-Natal
PEPFAR: The United States President’s Emergency Plan for AIDS Relief
PDA: Personal digital assistant
PHC: Primary health care
PMTCT: Prevention of mother-to-child transmission
UNAIDS: The Joint United Nations Programme on HIV/AIDS
VMMC: Voluntary medical male circumcision
